# Multiple Sclerosis Polygenic Risk Is Not Enriched in Three Multicase Families in Comparison to Population-Based Cases

**DOI:** 10.1155/2024/9268911

**Published:** 2024-05-16

**Authors:** Ming Chen, Allan Motyer, Bruce V. Taylor, Bennet J. McComish, Kathryn P. Burdon, Jac C. Charlesworth, Nicholas B. Blackburn

**Affiliations:** ^1^Menzies Institute for Medical Research, University of Tasmania, Hobart, Tasmania, Australia; ^2^Department of Clinical Laboratory, Chongqing Emergency Medical Center, Chongqing University Central Hospital, School of Medicine, Chongqing University, Chongqing 400014, China; ^3^Melbourne Integrative Genomics, The University of Melbourne, Melbourne, Victoria, Australia; ^4^School of Mathematics and Statistics, The University of Melbourne, Melbourne, Victoria, Australia; ^5^School of BioSciences, The University of Melbourne, Melbourne, Victoria, Australia

## Abstract

Multiple sclerosis (MS) is a complex neurological and autoimmune disease with an established genetic component. Families with multiple cases of MS are rare but do occur. We hypothesised that multicase families may have a heightened polygenic risk for MS. In this work, we have determined whether polygenic risk for MS is enriched in multicase families in comparison to a case-control cohort. Using the findings from the largest MS genome-wide association study, we calculated a weighted polygenic risk score (wPRS) for MS. We applied this wPRS to study a population-based MS case-control cohort (3,252 people with MS and 5,725 controls) and three multicase MS families (9 individuals with MS, 10 unaffected family members). For both the population-based cohort and the three families, 167 of the 233 known genome-wide significant MS-associated variants were identified and used to calculate the wPRS. Within the population-based cohort, the wPRS was significantly higher in MS cases than controls (*P* = 2.2 × 10^−16^). The wPRS of familial MS cases was not significantly different to population-based MS cases (*P* > 0.05). Both affected and unaffected MS family members had higher wPRS than population controls. MS families have a higher polygenic risk for MS, but this did not differ to the polygenic risk of population-based MS cases. Only one family carried the established *HLA-DRB1 15:01* MS risk allele, which was present in both affected and unaffected family members. Across families, unaffected family members had an elevated polygenic risk in comparison to population controls indicating that a higher polygenic risk does not fully explain the clustering of MS in families.

## 1. Introduction

The risk of developing multiple sclerosis (MS) has both genetic and environmental components [1]. Estimates of the genetic component range between 25% and 76%, depending on the study design [2–4]. A family history of MS is reported in 15%-20% of people with MS, and first degree relatives of people with MS have a 3% lifetime risk of developing MS themselves [5]. Families with three or more close relatives with MS are rare but well documented and provide opportunities, together with population-based case-control studies, to understand the genetic architecture of MS.

The largest genome-wide association study (GWAS) of MS to date analysed 47,429 people with MS and 68,374 control participants to identify 233 significant genetic common variant associations across the genome [6]. The strongest known genetic risk factor for MS is the *HLA-DRB1*∗*15:01* allele (odds ratio (OR) = 2.9, *P* = 1.62 × 10^−1916^) [6]. The common genetic architecture of MS captured by GWAS facilitates the construction of a polygenic risk score (PRS) for MS. A weighted PRS (wPRS) is a weighted sum aggregation of the total number of genetic risk associations an individual carries for a particular disease [7].

PRS have been previously derived and applied in MS using the genetic risk associations from earlier and smaller MS GWAS [8, 9]. More recent work using PRS derived from the largest MS GWAS to date [6] has shown gene-environment interactions between MS PRS and childhood obesity, a risk factor for MS development [10], and associations between an earlier age of MS onset and a higher PRS [11, 12]. Individuals in the highest quintile of PRS who also carry two copies of the *HLA-DRB1*∗*15:01* allele, on average, were 5 years younger at their age of MS onset [12]. Individuals that were in the highest decile of PRS were at a 5-fold increased risk of MS in a study of people with MS in the UK Biobank and a 15-fold higher risk in a smaller cohort of people with and without MS from the Kaiser Permanente in Northern California study [13]. A higher PRS, however, was not associated with the progression of MS disease from relapsing-remitting to secondary progressive disease [14]. Together, these studies show that an increased polygenic risk for MS may also influence components of the disease course.

Here, we calculate and compare a MS wPRS between MS cases from three multicase families to MS cases from a case-control population cohort. We hypothesised that an accumulation of polygenic risk for MS occurs in families and that families enriched for MS cases have an enriched burden of common MS risk variants in comparison to population cases.

## 2. Materials and Methods

### 2.1. ANZgene Case-Control Cohort

Baseline polygenic risk scores were calculated in a population relevant case-control cohort compiled by the Australia and New Zealand Multiple Sclerosis Genetics (ANZgene) Consortium and described in detail in Lin et al. [15]. All individual level and variant level quality control procedures and confirmation of European ancestry have been detailed previously [15]. The data were converted from hg18 coordinates to hg19 coordinates for analysis by matching SNP identifiers for the variants to the 1000 Genomes phase 3 reference dataset [16]. The final data included 3,252 people with MS and 5,725 controls, with genotyping data available for 271,943 autosomal SNPs (hg19).

### 2.2. Familial MS Cohort

Three MS families with at least three affected individuals were recruited for this study. Ethics approval was obtained from the Human Research Ethics Committee, Tasmania, Australia (H0026235), and written informed consent was obtained from all participating individuals. Families were ascertained by a MS neurologist (Professor Bruce Taylor), and family members with MS were diagnosed according to the 2017 McDonald criteria [17], with clinical histories obtained and reviewed for all family members diagnosed with MS. Unaffected relatives were over 50 years of age at recruitment and self-reported no clinical history of MS. DNA samples were available for nine individuals diagnosed with MS, eight unaffected relatives, and two unaffected parents of MS cases. All nine people with MS presented with relapsing-remitting MS at onset, with one individual converting to secondary progressive MS. All individuals underwent genotyping on the Illumina Infinium® Global Screening Array-24 v1.0. Data cleaning was conducted using the Illumina® GenomeStudio 2015 Software Genotyping Module v2.0.4 (Illumina, Inc.) and PLINK v1.90b6.10 [18] and is described further in the Supplementary Methods and Supplementary Table 1. Samples were confirmed to be of European ancestry through principal component analysis together with the European populations of the 1000 Genomes Project (Supplementary Figure 1) [16]. A final set of 560,702 variants (hg19) were used as input in this project.

### 2.3. Genome-Wide Imputation of the ANZgene Cohort and Familial MS Cohort

Genome-wide imputation of both the ANZgene cohort array data and the familial MS cohort array data was conducted separately. Genotypes were phased (Eagle v2.4) and imputed to the 1000 Genomes phase 3 version 5 dataset (European population) using Minimac4 and implemented using the Michigan Imputation Server [19], after exclusion of allelic mismatches between the reference panel and the genotyped data. For the ANZgene cohort, this resulted in 12,028,597 variants with an imputation quality score *R*^2^ ≥ 0.8 and for the familial MS cohort 7,442,746 variants with an imputation quality score *R*^2^ ≥ 0.8.

### 2.4. HLA Imputation

The HLA∗IMP:03 program was employed to conduct HLA-specific imputation from each array dataset [20]. Using the imputed SNP datasets, we extracted only the SNPs that intersected with the HLA∗IMP:03 training SNP dataset (HLA region on chromosome 6, 29.4 to 33.2 Mb, hg19). There were 676 SNPs from the imputed ANZgene cohort data and 2,466 SNPs from the imputed familial MS cohort data that intersected with the training dataset. These were provided as input to HLA∗IMP:03, and the classical four-digit HLA alleles were imputed. Amino acids for the HLA genes were mapped based on the corresponding HLA types using the HLA dictionary from the SNP2HLA package [21].

### 2.5. MS Risk Variant Selection

A list of the known common MS risk variants including 32 HLA variants and 200 autosomal risk SNPs was compiled [6]. For each imputed dataset, the known autosomal risk SNPs and HLA variants were extracted and then intersected to identify the MS risk variants available in both datasets. Ambiguous (A/T, C/G) SNPs were excluded from the analysis, resulting in exclusion of 4 HLA variants and 25 non-HLA variants. Of the known common variants, 26 (out of 32) HLA variants and 141 (out of 200) non-HLA variants were available in both datasets and were included in the analysis. Supplementary Figure 2 shows that the OR distributions of the variants that were included in comparison to those that were excluded were unbiased. Supplementary Table 2 summarises the MS risk variants that were available for analysis across both cohorts.

### 2.6. Weighted Polygenic Risk Score (wPRS) Calculation

We calculated the wPRS by using the allelic ORs from the MS GWAS data [6], to take the effect size of the risk alleles into account, as previously published [9]. In short, the wPRS of each individual was calculated by multiplying the number of copies of the risk allele for each SNP by the weighted effect of that SNP and summing across those values of all the SNPs. The weighted effect of a SNP was the natural log of the OR for the risk allele. The formula used to compute the wPRS is shown below.

(1) $$\begin{array}{c} \text{wPRS}=\overset{n}{\underset{i=1}{\sum }}G_{i}W_{i}, \end{array}$$

where *n* is the total number of risk alleles extracted from the study data, *i* is the risk allele, *G* is the number of copies (0, 1, or 2) of certain risk allele carried by an individual, and *W* is the weighted effect of a SNP, i.e., log(OR_*i*_).

### 2.7. Statistical Analysis

To test wPRS differences between the MS cases and the controls in the ANZgene cohort data, we performed a Welch two sample *t*-test. The receiver operating characteristic (ROC) curve was plotted to demonstrate the true positive rate against the false positive rate of using the wPRS to discriminate MS cases from controls. The area under the ROC curve (AUC) was calculated to assess the prediction performance of the wPRS in the case-control dataset.

To assess the difference between the cumulative common genetic burden in the familial MS cohort against the ANZgene cohort data, we again performed a Welch two sample *t*-test. As there is a sample size imbalance between the datasets, we iteratively subsampled the ANZgene cohort data 100 times generating random sets of either 500 MS cases or 500 controls which were then used to test against the familial MS cohort wPRS.

As a Welch two sample *t*-test cannot appropriately account for the nonindependence of the known relationships between family members, we also confirmed our main findings in a variant component framework using SOLAR (v8.4.2) [22, 23]. For comparisons of the familial data to the ANZgene cohort, this approach allowed a correlation matrix of relationships between family members to be included in a linear mixed model to appropriately test for wPRS differences in a related sample, while also controlling for sex. In these comparisons, the wPRS was inverse normalised to ensure a normal distribution.

## 3. Results

### 3.1. MS wPRS Distribution in a Case-Control Cohort (ANZgene)

**Figure 1:**
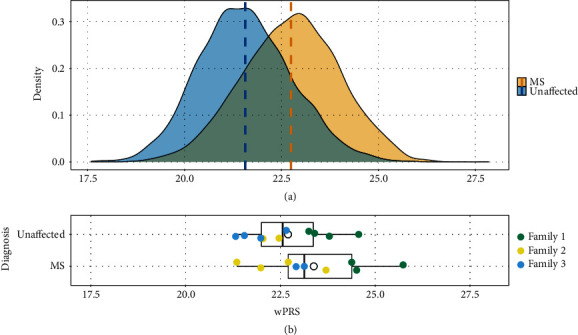
wPRS distribution in ANZgene case-control cohort and MS family study. (a) A density plot showing the distribution of wPRS in the ANZgene case-control cohort. Vertical dashed lines indicate the mean value for each distribution. (b) Boxplots representing the distribution of wPRS values for the unaffected and affected individuals in the MS families; the mean within each boxplot is shown by the white circle. Points are colour coded by family and separated by disease status.

In the ANZgene case-control cohort, the mean wPRS was significantly higher in MS cases (mean = 22.75, SD = 1.25) compared with controls (mean = 21.57, SD = 1.20; *P* = 2.2 × 10^−16^) and demonstrated an overall right-skewed distribution (inflation) of wPRS in the MS cases compared to the controls (Figure 1(a)). The wPRS computed based on the 26 HLA variants, and the 141 non-HLA variants exhibited a moderate capacity to discriminate between MS patients and controls in the ANZgene cohort (AUC = 0.75, 95% confidence interval (CI): 0.74–0.76; Supplementary Figure 3).

### 3.2. wPRS Characteristics in Familial MS

**Figure 2:**
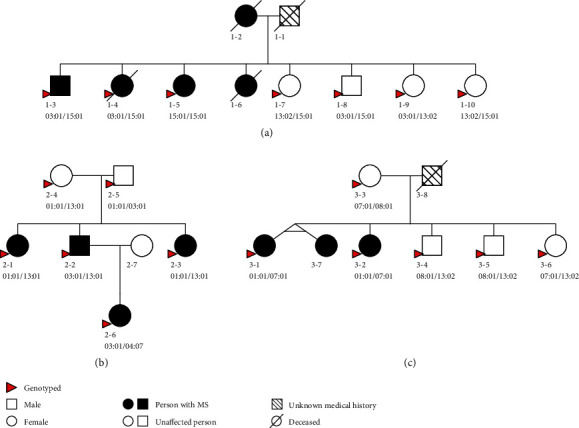
Pedigree diagrams of the three families showing *HLA-DRB1* alleles: (a) Family 1; (b) Family 2; (c) Family 3. Individuals 3-1 and 3-7 are joined as monozygotic twins. The pedigree diagram was constructed using CraneFoot v3.2 [40]. Individuals with available genotype data are indicated with red arrows. Beneath each individual's pedigree symbol is their ID, followed by their *HLA-DRB1* allele genotypes depicting the segregation of the alleles at this locus within each family and the occurrence of the *HLA-DRB1 15:01* MS risk allele in Family 1.

Calculation of the wPRS in the familial MS cohort identified a range of polygenic risk for MS across people with and without MS and notable differences between families. Family-specific wPRS distributions and values are presented in Figure 1(b) and Table 1.  Table 2 summarises the individual-level wPRS in the three families and identifies carriers of the *HLA-DRB1*∗*15:01* risk allele. For individuals in Family 1, there was an overall higher wPRS than in Family 2 or Family 3 with the average wPRS in Family 1 unaffected individuals (mean = 23.75, SD = 0.59) exceeding the scores for the people with MS in both Family 2 (mean = 22.44, SD = 1.01) and Family 3 (mean = 23.02, SD = 0.15). In Family 1, all genotyped siblings share the *HLA-DRB1*∗*15:01* allele (five individuals were heterozygous, individual 1-5 was homozygous), except for one unaffected relative 1-9, who inherited the alternate *HLA-DRB1*∗*03:01* and *HLA-DRB1*∗*13:02* allele from their parents (Figure 2(a)). It is worth noting that even though individual 1-9 does not carry *HLA-DRB1*∗*15:01*, their wPRS is still higher than most affected individuals (except individual 2-6) of the other two families (Table 2). No other HLA MS risk alleles were observed in any of the three families (Figure 2).

The absence of other HLA MS risk alleles in either Family 2 or Family 3 (Figure 2) highlights the underlying difference in the genetic architectures of these three MS families. In Family 2, the wPRS is variable with no clear pattern between generations. In comparison to the distribution of the wPRS in the ANZgene population controls (Figure 1(a)), all family members in the MS families (Figure 1(b)) display a right-skewed wPRS distribution. Family 1 has the most extreme wPRS distribution with both affected and unaffected individuals showing wPRS values above the mean of the ANZgene MS cases. The wPRS by comparison for both affected and unaffected individuals in Family 2 and Family 3 are spread between the means of the ANZgene controls and the ANZgene cases.

### 3.3. Impact of Common Genetic Risk on MS Clustering in Families

To identify whether there is an enrichment in polygenic risk for MS among individuals with a strong family history of MS, we compared the wPRS calculated in the familial MS cohort with the ANZgene cohort data. We randomly subsampled 500 MS cases or 500 controls from the ANZgene cohort to compare with the smaller cohort of familial MS cases or their unaffected relatives. On average across 100 subsampling iterations, familial MS cases had higher wPRS (mean = 23.38, SD = 1.36) than that of ANZgene cohort controls (subsampling mean = 21.62, subsampling SD = 1.22). The higher wPRS in familial MS cases in comparison to ANZgene cohort controls was significant across each of the 100 subsampling iterations (minimum *P* value = 0.002, maximum *P* value = 0.007). No significant difference was observed between the wPRS of familial MS cases and ANZgene cohort cases (*P* > 0.05 in all iterations). This indicated that the wPRS of the familial MS cases was neither higher nor lower than the ANZgene cohort cases, providing evidence that the polygenic risk of MS is not substantially different to unrelated MS cases from the population. Finally, we identified that the wPRS of unaffected relatives (mean = 22.70, SD = 1.04) in the familial MS cohort in comparison to the ANZgene cohort controls (subsampling mean = 21.59, subsampling SD = 1.14) was statistically significantly higher across all subsampling iterations (minimum *P* value = 0.004, maximum *P* value = 0.01). This provides evidence that the polygenic risk for MS is elevated in these families, even among unaffected family members, but indicates that this alone is insufficient to lead to the development of MS.

Evaluation of these comparisons using SOLAR to account for the known genetic relationships within the MS families, control for sex, and include all ANZgene cohort cases or controls in the same comparisons identified consistent associations. Familial MS cases had a higher wPRS than ANZgene cohort controls (*P* = 0.001, *β* = 1.51 standard deviation units higher). No significant difference was observed between the wPRS of familial MS cases and ANZgene cohort cases (*P* = 0.21). Unaffected relatives in comparison to all ANZgene cohort controls had a higher wPRS (*β* = 0.85 standard deviation units higher). However, this did not surpass a nominal significance threshold (*P* = 0.052) but was consistent with direction of effect.

## 4. Discussion

In this study, we calculated a MS wPRS in a population-based MS case control and in three multicase families. The population-level wPRS followed a normal distribution, which is in agreement with the theories of Clayton [24] and Pharoah et al. [25], supporting the fact that MS is a complex disease determined by multiple susceptibility variants. As expected, the wPRS was significantly higher in MS cases than controls (*P* = 2.2 × 10^−16^). A model using this wPRS exhibited a moderate predictive capacity to discriminate between MS cases and controls (AUC = 0.75, 95% CI: 0.74–0.76). These results are comparable with earlier PRS studies in MS, where the inclusion of the additional variants moderately improved performance of the wPRS in predicting high-risk individuals [9, 26–29]. However, the predictive capacity is still limited in clinical practice for MS [30], as a higher specificity would be required for the accurate prediction of MS. One limitation here is that both cases and controls from this dataset contributed to published MS GWAS that the PRS variants are derived from. Therefore, it was expected that there would be PRS differences between MS cases and controls in this dataset. It was necessary to establish this difference to continue with our main analysis of comparing the PRS from MS families to the PRS distribution in the population-based dataset. The second limitation of this work is that difference in array-based technologies across datasets, despite genotype imputation, meant that not all genome-wide significant variants for MS risk were able to be included in the wPRS calculation. Of the published 233 MS risk variants, 26 out of 32 HLA variants and 141 out of 201 non-HLA variants were available in both the family and cohort datasets and were included in the analysis. All variants with ORs ≥ 2 were included in the wPRS calculation, and the maximum OR of variants that could not be included was 1.589.

We hypothesised that there is an accumulation of common polygenic MS risk variants in MS families and that familial MS cases have a higher wPRS than population cases. While the familial MS cases had a higher wPRS than population controls (minimum *P* value = 0.002, maximum *P* value = 0.007), there was no statistical difference in the wPRS between familial MS cases and population-based MS cases (*P* > 0.05). This suggests that familial MS cases do have an accumulation of common polygenic MS risk, but this is no greater than population-based MS cases. Even unaffected relatives of familial MS cases had a higher wPRS than population controls (minimum *P* value = 0.004, maximum *P* value = 0.01). Together, this suggests that families with multiple cases of MS do have a higher wPRS than unaffected individuals in the population, but this may not fully explain the clustering of MS cases within these families, with unaffected relatives also having higher polygenic risk. Previous studies have identified conflicting results in this area, with some studies observing enrichment of risk variants in families [28, 29] and others showing similar results to our data [27, 31]. A limitation of these previous studies was that they were conducted prior to the latest MS GWAS and thus used fewer MS risk variants to compute their polygenic risk scores. Recent work in a Turkish cohort has suggested that population-based MS cases have a higher PRS than people with MS from multicase families; however, this comparison was not statistically significant, and not all HLA risk alleles were able to be imputed [32]. These conflicting results also reflect the heterogeneity of common genetic risk profiles for MS. This is consistent with the per family variation we observed, especially where the presence or absence of major risk alleles (i.e., *HLA-DRB1*∗*15:01*) can have a large impact on overall scores.

At the individual family level, we identified a spectrum of polygenic risk. The largest common variant risk association from MS GWAS, the *HLA-DRB1*∗*15:01* allele, only occurred in one of the three families (Family 1). This allele was also present in three of the four unaffected siblings in this family, indicating that alone it does not explain the MS clustering in this family. No other MS-associated HLA risk variants were observed in these families. Therefore, we propose that other variants of large effect size are likely to be shared among the affected family members, or alternatively, protective variant(s) may be present in the unaffected relatives.

Although there were a small number of risk variants not captured in this study, the OR effect sizes of these missing variants are unbiased with respect to the variants that were available (Supplementary Figure 2). Thus, the results of this study should be generally indicative of the wider common variant risk for MS. While the wPRS is based on the largest set of common risk loci for MS to date, other common risk variants may exist. However, the chance of identifying novel common variants in European populations with even moderate effects for complex diseases by conducting additional GWASs is low [33, 34]. A limitation of the current study is that the individual cases and controls within the ANZgene cohort contributed to the 2019 GWAS study [6] from which the MS risk variants were drawn to form the wPRS used here. Thus, the population cohort in this study does not represent an independent sample to the previous MS GWAS. The population cohort is used here to set the background of polygenic risk for MS on which we evaluate the familial MS cohort.

Our observation that unaffected relatives of familial MS cases exhibited a higher wPRS than population controls indicates that they carry genetic burden similar to their affected family members. However, they have not developed MS. This suggests that the known common MS risk variants are not sufficient to drive the MS clustering in these families, and we hypothesise that the familial aggregation of MS is likely to be driven by a combination of a common variants as well as multiple rare or family-specific variants. Others have also postulated that part of the heritability of complex diseases such as MS may be due to rare variants [35–37] and multiple studies have demonstrated the impact of rare variants on complex disease such as Alzheimer's disease [38].

Considerable opportunity remains for the identification of MS genes in the genomics era through the study of multicase MS families, potentially leading to biologically impactful genetic understandings of the disease.

## Figures and Tables

**Table 1:** Mean wPRS results in each of the three MS families by affection status.

Family	Affected *μ* wPRS (SD)	Unaffected *μ* wPRS (SD)
1	24.88 (0.75)	23.75 (0.59)
2	22.44 (1.01)	22.25 (0.30)
3	23.02 (0.15)	21.88 (0.59)
Combined	23.38 (1.36)	22.70 (1.04)

SD = standard deviation. The wPRS of affected and unaffected individuals from each family were summed to represent the combined overall mean wPRS and SD by group.

**Table 2:** Individual MS wPRS and HLA-DRB1∗15:01 risk allele status in the MS families.

Family	Individual ID	MS status^#^	wPRS	*HLA-DRB1*∗*15:01^##^*
1	1-3	+	24.38	+/-
	1-4	+	24.51	+/-
	1-5	+	25.74	+/+
	1-7	-	23.40	+/-
	1-8	-	23.79	+/-
	1-9	-	23.25	-/-
	1-10	-	24.56	+/-

2	2-1	+	21.35	-/-
	2-2	+	21.98	-/-
	2-3	+	22.71	-/-
	2-6	+	23.70	-/-
	2-4	-	22.04	-/-
	2-5	-	22.46	-/-

3	3-1	+	23.13	-/-
	3-2	+	22.91	-/-
	3-3	-	21.98	-/-
	3-4	-	21.32	-/-
	3-5	-	21.55	-/-
	3-6	-	22.66	-/-

^#^MS status: person with MS (+), unaffected person (-). ^##^*HLA-DRB1*∗*15:01*: individuals carry zero (-/-), one (+/-), or two (+/+) copies of allele.

## Data Availability

All data produced in the present study are available upon reasonable request, for work consistent with the original purpose of the data, to the corresponding author.

## Supplementary Materials

Supplementary Materials The supplementary material file contains additional methodological details as to the preparation and processing of the genotype array data from the family-based samples (supplementary methods and supplementary table 1) including a principal component analysis confirmation of European ancestry (supplementary figure 1). Supplementary figure 2 compares the published effect sizes of MS risk variants that were included in the wPRS calculation to those that were not able to be identified in the dataset. Supplementary table 2 lists each variant included in the wPRS calculation.
