# Supplementary material for: Multiple Sclerosis Polygenic Risk Is Not Enriched in Three Multicase Families in Comparison to Population-Based Cases
Source: Hum Mutat. 2024 May 16;2024:9268911. doi: 10.1155/2024/9268911 (PMC11919224; doi:10.1155/2024/9268911)
Supplement: Supplementary Materials — The supplementary material file contains additional methodological details as to the preparation and processing of the genotype array data from the family-based samples (supplementary methods and supplementary table 1) including a principal component analysis confirmation of European ancestry (supplementary figure 1). Supplementary figure 2 compares the published effect sizes of MS risk variants that were included in the wPRS calculation to those that were not able to be identified in the dataset. Supplementary table 2 lists each variant included in the wPRS calculation. [file 9268911.f1.docx]

**Supplementary Methods**

Illumina Infinium^®^ Global Screening Array-24 v1.0 (GSA) data processing of familial MS cohort data

The raw intensity data were loaded into GenomeStudio to assign genotype calls. Sex estimates were calculated from the data and confirmed to be consistent with recorded sex for each sample. Autosomal SNPs were filtered and excluded based on the following criteria: SNPs with call frequency < 1; SNPs with AB R Mean less than 0.5 (intensities of the SNPs were too low to call genotypes); SNPs with AB T Mean less than 0.2 (SNPs where the heterozygote cluster has shifted toward the left homozygote cluster) or greater than 0.8 (SNPs where the heterozygote cluster has shifted toward the right homozygote cluster); SNPs with either parent-child (P-C) errors > 0 or parent-parent-child (P-P-C) errors > 0 (these were used to identify SNPs with deviations from the expected parent-child and father-mother-child heritability patterns). Other types of markers, which include mitochondrial DNA (mtDNA), chrY and chrXY SNPs (the pseudoautosomal SNPs on sex chromosomes) were also excluded. Finally, four filters were applied to remove poorly clustered calls, as recommended by the technical note: A/A frequency = 1 and AA T Mean > 0.3; A/A frequency = 1 and AA T Dev > 0.06; B/B frequency = 1 and BB T Mean < 0.7 and B/B frequency = 1 and BB T Dev > 0.06.

After data cleaning in GenomeStudio, we performed standard QC steps (Anderson et al. 2010) for the array data from the family samples using PLINK v1.90b6.10 (Chang et al. 2015). No samples were filtered based on a missing call rate threshold of > 3% or were outliers for heterozygosity rates (deviation from the mean ± 2 standard deviations, SD). For marker-level QC, all the SNPs were filtered based on the missing call rates (> 1%) and differential missingness between the familial MS cases and familial MS controls (*P* < 0.001). Supplementary Table 1 summarises the variant exclusions.

**Supplementary Table 1 Quality control of GSA data**

| **Filter condition** | **Number of variants retained** | **Number of variants excluded** |
| --- | --- | --- |
| Call frequency < 1 | 591,812 | 28,356 |
| AB R Mean < 0.5 | 569,348 | 22,464 |
| AB T Mean (< 0.2) or (>0.8) | 568,834 | 514 |
| P-C errors > 0 | 566,717 | 489 |
| P-P-C errors > 0 | 566,207 | 510 |
| Male heterozygous chrX | 566,195 | 12 |
| Other markers (mtDNA, chrY, chrXY SNPs) | 564,389 | 1,806 |
| A/A_frequency = 1 and AA T Mean > 0.3 | 564,345 | 44 |
| A/A_frequency = 1 and AA T Dev > 0.06 | 564,345 | 0 |
| B/B_frequency = 1 and BB T Mean < 0.7 | 564,118 | 227 |
| B/B_frequency = 1 and BB T Dev > 0.06 | 564,109 | 9 |
| Single base Indels | 561,932 | 2,177 |
| Triallelic SNPs | 560,751 | 1,181 |
| Missingness > 1% | 560,702 | 49 |
| **Initial variants:** 618,540  **Cleaned variants:** 560,702 | | |

Confirmation of European ancestry using principal components analysis

A principal components analysis (PCA) was conducted in PLINK (v1.90b6.10) using data from the European populations of the 1000 genomes project (The 1000 Genomes Project Consortium et al. 2015) to validate the European descent of all participants. The 560,702 array variants were intersected with the European ancestry samples of the 1000 genomes project phase 3 data release. An overlapping set of 497,659 variants was identified which were LD pruned. To generate a final set of 18,696 variants for PCA analysis. Supplementary figure 1 shows the PCA analysis results with all study samples clustering with the CEU and GBR European 1000 genomes project populations.

**Supplementary Figure 1**

**
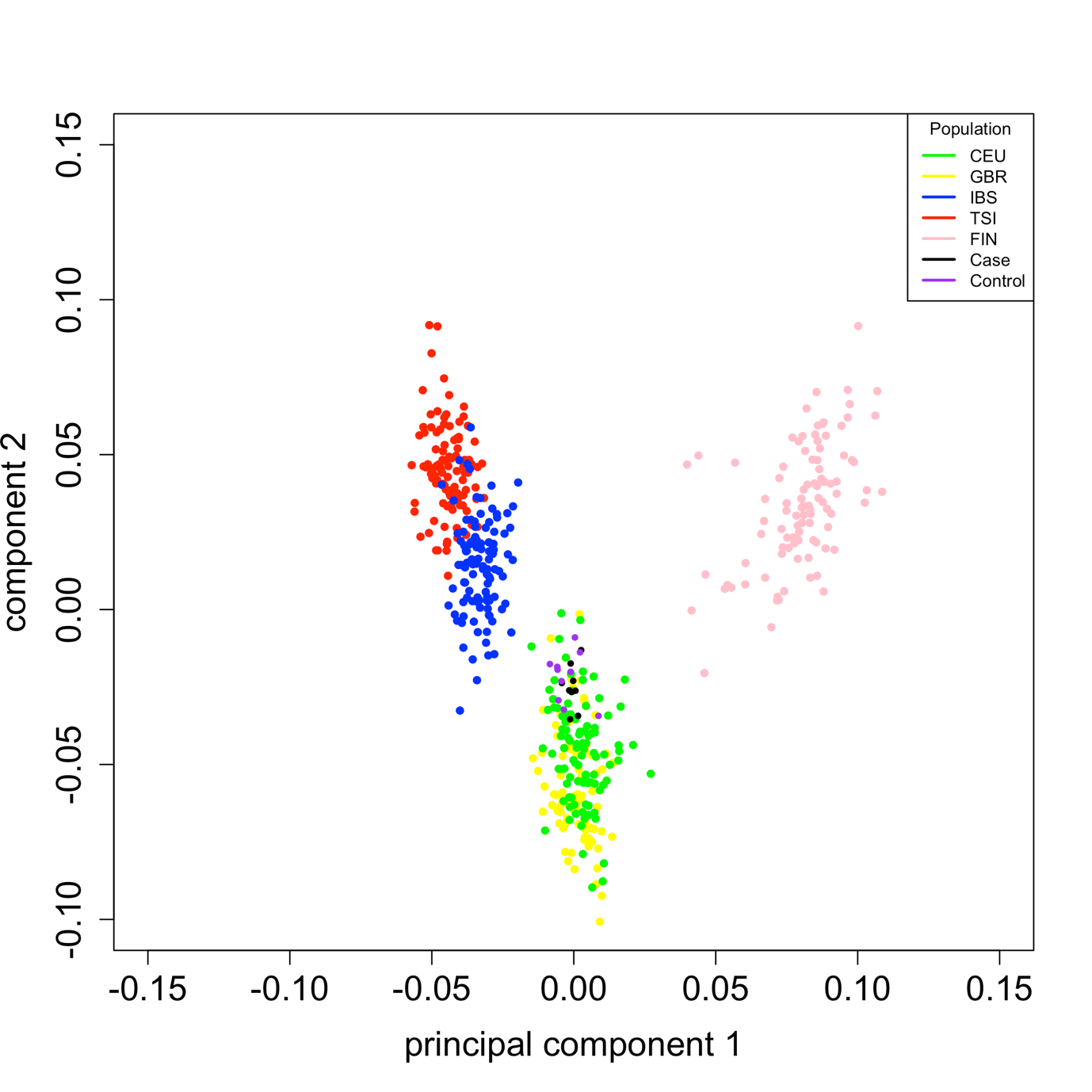
**

**Principal Component Analysis of familial MS cohort study samples with 1000 genomes project European population data.** A principal component analysis of the familial MS cohort data together with European population data from the 1000 genomes project confirms that the familial samples (‘Case’ and ‘Control’) are of European ancestry, clustering with the CEU and GBR subpopulations.

**Supplementary Figure 2**

**
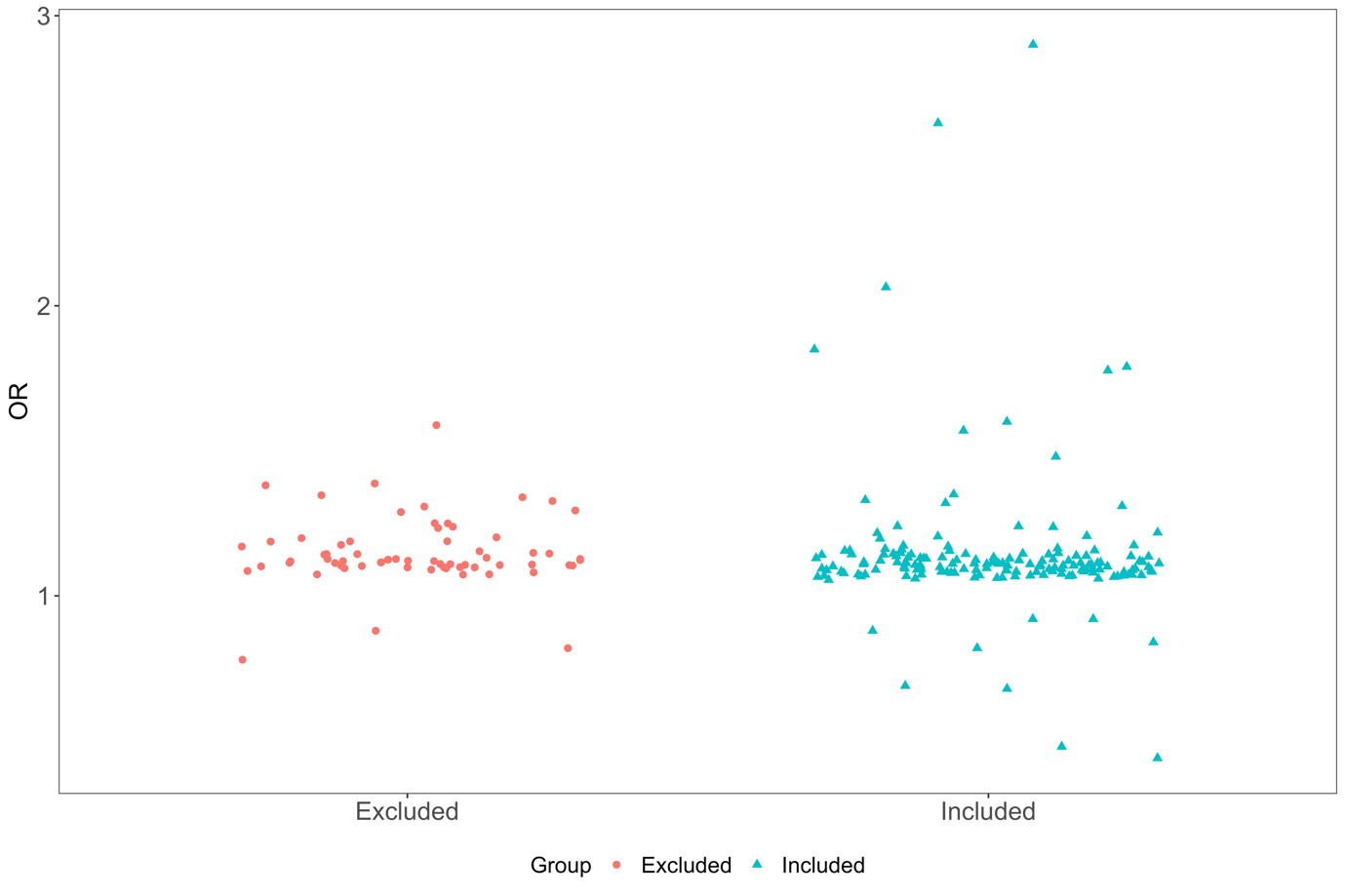
**

**Comparison of OR effect sizes of variants included and excluded in the wPRS**

Scatter plot showing the distribution of ORs of MS risk variants (from (International Multiple Sclerosis Genetics Consortium 2019)) that were excluded (red) from the wPRS calculation because of variant ambiguity or mismatch between datasets, in comparison to those that were included (blue). The ORs of those excluded variants are small and unbiased in the distribution of the overall ORs. In contrast, the relatively large ORs are all in those variants that were included in the study. The average OR of the included variants was 1.143 (SD = 0.248), compared with that of the excluded variants was 1.150 (SD = 0.120). All the variants with ORs ≥ 2 were included in this study, while the maximum OR of the excluded variant is 1.589.

**Supplementary Figure 3**


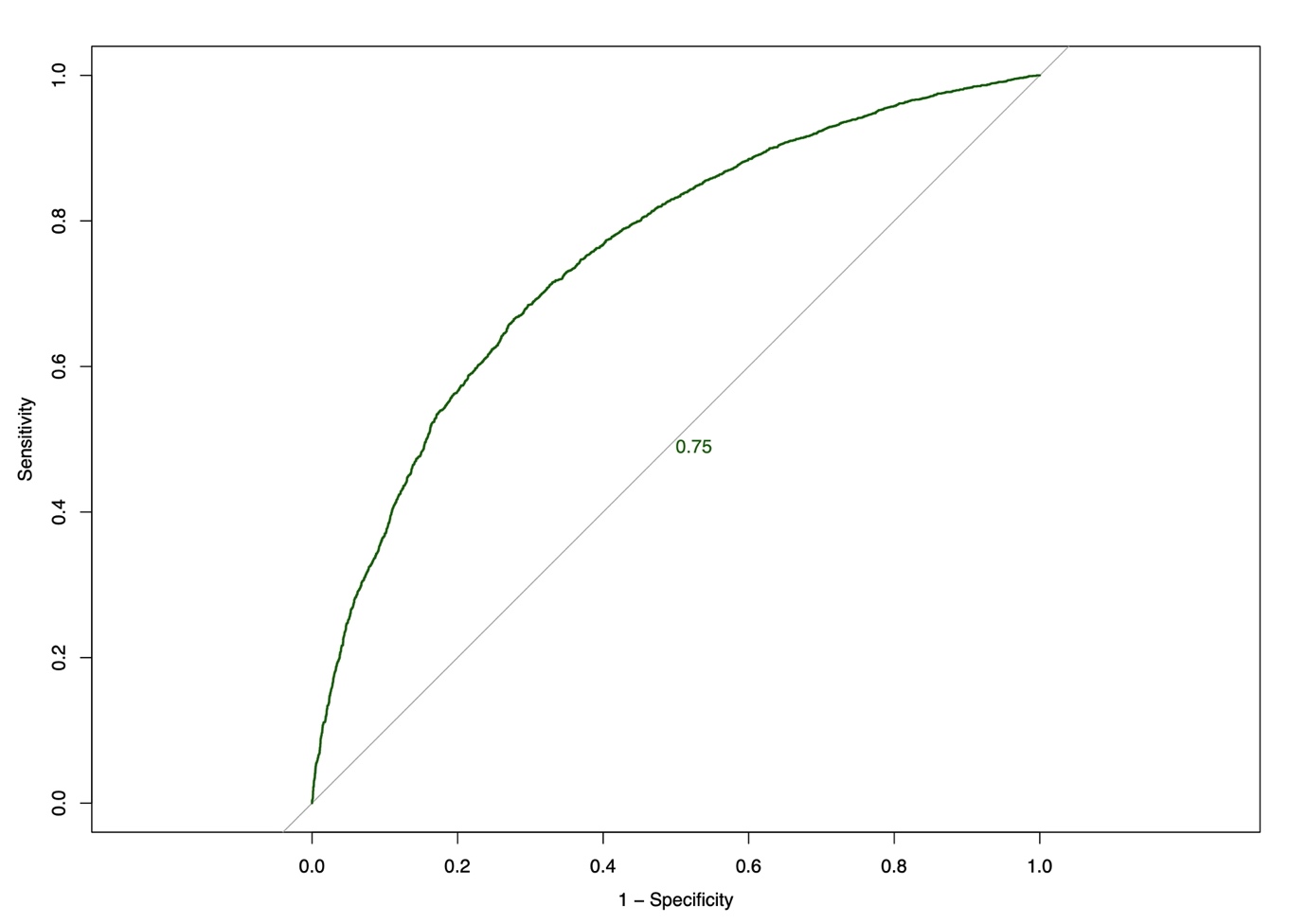


**ROC curve for MS prediction.**

Receiver Operating Characteristic (ROC) curve corresponding to wPRS based on 26 HLA and 141 non-HLA common variants. The area under the ROC curve (AUC) was 0.75 providing a measure of the prediction performance of the wPRS in discriminating people with MS and controls in the ANZgene cohort.

**Supplementary Table 2 MS risk variants included in this study**

| **chromosome** | **position** | **affect_allele** | **non_affect_allele** | **odds_ratio** |
| --- | --- | --- | --- | --- |
| 1 | 101290432 | A | G | 1.218 |
| 1 | 101412902 | G | T | 1.094 |
| 1 | 117090493 | C | T | 1.24 |
| 1 | 157686337 | G | T | 1.106 |
| 1 | 160389984 | A | G | 1.091 |
| 1 | 160634588 | C | T | 1.112 |
| 1 | 160703965 | G | A | 1.107 |
| 1 | 192541021 | A | G | 1.132 |
| 1 | 200875897 | T | C | 1.131 |
| 1 | 212877776 | G | A | 1.096 |
| 1 | 2520527 | T | C | 1.156 |
| 1 | 32738415 | A | G | 1.145 |
| 1 | 6512547 | C | T | 1.112 |
| 1 | 65429319 | G | A | 1.141 |
| 1 | 85682020 | G | A | 1.091 |
| 1 | 85729820 | G | A | 1.197 |
| 1 | 92222089 | C | T | 1.137 |
| 1 | 92939959 | C | T | 1.104 |
| 1 | 93152635 | T | C | 1.155 |
| 10 | 6070273 | C | A | 1.145 |
| 10 | 6117322 | T | C | 1.205 |
| 10 | 64449549 | G | T | 1.115 |
| 10 | 75653800 | G | T | 1.093 |
| 10 | 8098719 | C | T | 1.083 |
| 10 | 81059335 | T | G | 1.123 |
| 10 | 94479107 | A | G | 1.111 |
| 11 | 118743286 | G | A | 1.155 |
| 11 | 118747813 | A | G | 1.109 |
| 11 | 122518525 | G | A | 1.078 |
| 11 | 128421175 | G | A | 1.069 |
| 11 | 47360412 | C | T | 1.09 |
| 11 | 64095178 | C | T | 1.076 |
| 11 | 65705432 | T | C | 1.092 |
| 11 | 95311422 | C | T | 1.094 |
| 12 | 111884608 | T | C | 1.071 |
| 12 | 123604053 | T | C | 1.129 |
| 12 | 6440009 | C | T | 1.135 |
| 12 | 6514963 | A | C | 1.099 |
| 12 | 94661453 | G | A | 1.083 |
| 13 | 100026952 | A | C | 1.777 |
| 13 | 50811220 | T | C | 1.237 |
| 14 | 103265844 | A | G | 1.104 |
| 14 | 52306091 | C | A | 1.101 |
| 14 | 76014298 | T | C | 1.116 |
| 15 | 79247482 | C | T | 1.133 |
| 15 | 90887584 | C | A | 1.106 |
| 16 | 1067832 | T | G | 1.126 |
| 16 | 11114512 | A | G | 1.115 |
| 16 | 11213951 | C | T | 1.206 |
| 16 | 11353879 | T | C | 2.064 |
| 16 | 11412926 | C | A | 1.123 |
| 16 | 30103160 | C | A | 1.102 |
| 16 | 57077094 | T | C | 1.089 |
| 16 | 79111297 | C | T | 1.099 |
| 17 | 34842521 | G | A | 1.089 |
| 17 | 37970149 | G | A | 1.074 |
| 17 | 40529835 | A | G | 1.138 |
| 17 | 45702280 | C | T | 1.113 |
| 17 | 57859210 | G | A | 1.109 |
| 18 | 56269737 | A | G | 1.06 |
| 18 | 67544046 | C | T | 1.063 |
| 19 | 10592144 | T | C | 1.138 |
| 19 | 11173928 | C | T | 1.084 |
| 19 | 16559421 | G | A | 1.095 |
| 19 | 45143942 | C | A | 1.093 |
| 19 | 49837246 | T | C | 1.132 |
| 19 | 6668972 | C | T | 1.164 |
| 2 | 112770799 | G | A | 1.128 |
| 2 | 12607893 | C | T | 1.109 |
| 2 | 136884679 | T | C | 1.102 |
| 2 | 151644203 | C | T | 1.07 |
| 2 | 191989356 | C | A | 1.068 |
| 2 | 231121829 | C | T | 1.145 |
| 2 | 25052177 | C | T | 1.062 |
| 2 | 61242410 | G | A | 1.113 |
| 2 | 65661843 | C | T | 1.072 |
| 2 | 68646536 | T | C | 1.113 |
| 20 | 39968188 | A | G | 1.079 |
| 20 | 42579051 | C | T | 1.07 |
| 20 | 44734310 | C | T | 1.143 |
| 20 | 52744437 | C | T | 1.137 |
| 20 | 62374441 | C | T | 1.173 |
| 21 | 34787312 | G | A | 1.085 |
| 22 | 22205353 | T | C | 1.121 |
| 22 | 37258986 | C | T | 1.088 |
| 22 | 37310954 | A | G | 1.06 |
| 22 | 40291807 | T | C | 1.065 |
| 22 | 50971266 | T | C | 1.117 |
| 3 | 100848597 | C | T | 1.162 |
| 3 | 101749022 | C | T | 1.104 |
| 3 | 105455955 | C | T | 1.089 |
| 3 | 119228508 | G | A | 1.143 |
| 3 | 121542898 | C | T | 1.089 |
| 3 | 141150990 | G | A | 1.067 |
| 3 | 159691112 | C | T | 1.111 |
| 3 | 169536637 | T | C | 1.115 |
| 3 | 187565968 | G | T | 1.055 |
| 3 | 187987624 | C | T | 1.072 |
| 3 | 18798848 | C | T | 1.068 |
| 3 | 27783015 | T | C | 1.12 |
| 3 | 28072086 | C | T | 1.148 |
| 3 | 32962051 | A | G | 1.073 |
| 3 | 71535338 | T | G | 1.087 |
| 4 | 103911781 | A | G | 1.067 |
| 4 | 106255589 | C | T | 1.082 |
| 4 | 109058718 | G | T | 1.094 |
| 4 | 122119449 | T | C | 1.099 |
| 4 | 164493807 | G | A | 1.07 |
| 4 | 40307564 | C | T | 1.073 |
| 4 | 48127262 | G | A | 1.09 |
| 5 | 118703662 | T | G | 1.071 |
| 5 | 133891282 | C | T | 1.083 |
| 5 | 141539339 | A | C | 1.08 |
| 5 | 158759900 | A | G | 1.124 |
| 5 | 176790162 | G | A | 1.1 |
| 5 | 35877505 | G | A | 1.104 |
| 5 | 40396425 | A | G | 1.217 |
| 5 | 55444683 | G | A | 1.097 |
| 5 | 6712834 | C | T | 1.084 |
| 6 | 33081632 | G | A | 1.17 |
| 6 | 32552064 | P | A | 1.6 |
| 6 | 119215402 | A | C | 1.083 |
| 6 | 32586854 | G | A | 1.57 |
| 6 | 32608306 | P | A | 1.85 |
| 6 | 128280104 | T | C | 1.121 |
| 6 | 135495226 | T | C | 1.125 |
| 6 | 137438057 | A | G | 1.147 |
| 6 | 137959455 | A | C | 1.116 |
| 6 | 143865221 | G | A | 1.157 |
| 6 | 31323293 | P | A | 0.44 |
| 6 | 33047466 | C | T | 1.16 |
| 6 | 159465977 | C | T | 1.12 |
| 6 | 31518354 | C | T | 0.92 |
| 6 | 32632795 | P | A | 0.88 |
| 6 | 16672760 | T | C | 1.067 |
| 6 | 32552138 | P | A | 0.84 |
| 6 | 31323293 | P | A | 1.33 |
| 6 | 33081823 | G | A | 0.92 |
| 6 | 32551948 | P | A | 1.32 |
| 6 | 32552064 | P | A | 2.9 |
| 6 | 31539767 | A | G | 1.35 |
| 6 | 31322522 | A | C | 1.09 |
| 6 | 31346822 | C | T | 0.82 |
| 6 | 36348689 | T | C | 1.079 |
| 6 | 32552064 | P | A | 2.63 |
| 6 | 29911056 | P | A | 0.69 |
| 6 | 31323293 | P | A | 0.48 |
| 6 | 31497244 | T | C | 1.11 |
| 6 | 32629138 | P | A | 0.68 |
| 6 | 31323677 | T | C | 1.31 |
| 6 | 32552064 | P | A | 1.79 |
| 6 | 90976768 | A | G | 1.174 |
| 6 | 31324602 | P | A | 1.13 |
| 6 | 32634302 | P | A | 1.24 |
| 6 | 32609875 | P | A | 1.48 |
| 7 | 128573967 | A | G | 1.063 |
| 7 | 138729795 | A | C | 1.073 |
| 7 | 149289464 | G | A | 1.114 |
| 7 | 28142186 | T | G | 1.065 |
| 7 | 3139417 | C | T | 1.082 |
| 7 | 37382465 | G | T | 1.143 |
| 8 | 128175696 | A | G | 1.089 |
| 8 | 128814091 | G | A | 1.113 |
| 8 | 129177769 | C | T | 1.129 |
| 8 | 144986793 | T | C | 1.073 |
| 8 | 79417222 | G | A | 1.143 |
| 9 | 100868189 | T | C | 1.116 |

**Supplementary References**

Anderson, C. A., F. H. Pettersson, G. M. Clarke, L. R. Cardon, A. P. Morris, and K. T. Zondervan. 2010. "Data quality control in genetic case-control association studies." *Nat Protoc* 5 (9): 1564-73. <https://doi.org/10.1038/nprot.2010.116>. <https://www.ncbi.nlm.nih.gov/pubmed/21085122>.

Chang, C. C., C. C. Chow, L. C. Tellier, S. Vattikuti, S. M. Purcell, and J. J. Lee. 2015. "Second-generation PLINK: rising to the challenge of larger and richer datasets." *Gigascience* 4: 7. <https://doi.org/10.1186/s13742-015-0047-8>. <https://www.ncbi.nlm.nih.gov/pubmed/25722852>.

International Multiple Sclerosis Genetics Consortium. 2019. "Multiple sclerosis genomic map implicates peripheral immune cells and microglia in susceptibility." *Science* 365 (6460). <https://doi.org/10.1126/science.aav7188>. <https://www.ncbi.nlm.nih.gov/pubmed/31604244>.

The 1000 Genomes Project Consortium, A. Auton, L. D. Brooks, R. M. Durbin, E. P. Garrison, H. M. Kang, J. O. Korbel, J. L. Marchini, S. McCarthy, G. A. McVean, and G. R. Abecasis. 2015. "A global reference for human genetic variation." *Nature* 526 (7571): 68-74. <https://doi.org/10.1038/nature15393>. <https://www.ncbi.nlm.nih.gov/pubmed/26432245>.
